# Cardiac arrest in a patient with anterior fascicular block after administration of dexmedetomidine with spinal anesthesia: A case report

**DOI:** 10.1097/MD.0000000000005278

**Published:** 2016-10-28

**Authors:** Baek Jin Kim, Bong Il Kim, Sung Hye Byun, Eugene Kim, Shin Yeung Sung, Jin Yong Jung

**Affiliations:** Department of Anesthesiology and Pain Medicine, School of Medicine, Catholic University of Daegu, Daegu, Republic of Korea.

**Keywords:** anterior fascicular block, bradycardia, cardiac arrest, dexmedetomidine

## Abstract

**Background:**

Dexmedetomidine is a sedative and analgesic agent that is administered intravenously as an adjunct to spinal anesthesia. It does not suppress the respiratory system significantly, but has adverse effects on the cardiovascular system, for example, bradycardia and hypotension. We here report a patient who underwent cardiac arrest during spinal anesthesia after intravenous infusion of dexmedetomidine.

**Methods:**

A 57-year-old woman with no significant medical history underwent spinal anesthesia for arthroscopic meniscus resection after rupturing the right knee meniscus. Preoperative electrocardiogram revealed sinus bradycardia (54 beats/min) and a left anterior fascicular block. Spinal anesthesia was performed with 11 mg of 0.5% heavy bupivacaine, and the upper level of sensory loss was at T6. Dexmedetomidine infusion was planned at a loading dose of 1.0 mcg kg^−1^ min^−1^ over 10 minutes, followed by 0.7 mcg kg^−1^ min^−1^ intravenously, as a sedative. Two minutes after dexmedetomidine injection, her heart rate decreased to 31 beats/min and asystole was observed within 10 seconds.

**Results:**

After a few minutes of cardiopulmonary resuscitation, spontaneous circulation returned and surgery was completed under general anesthesia. The patient was discharged, and experienced no complications.

**Conclusion:**

Dexmedetomidine can decrease blood pressure and heart rate, and may cause asystole in some cases. We suggest that dexmedetomidine should be carefully administered under close observation when the parasympathetic nerve system is activated during spinal anesthesia.

## 1 Introduction

Dexmedetomidine is an α-2 agonist that has sedative and analgesic effects, but does not cause severe suppression of the respiratory system. It is used as an adjuvant drug to general anesthesia or regional anesthesia. In particular, when it is used to supplement regional anesthesia, it prolongs the duration of the sensory and motor block by approximately 200 minutes^[1]^ and reduces the postoperative requirement for analgesics. Dexmedetomidine exerts its properties of relaxation of tension, sedation, and analgesia by acting on the central nervous system. It inhibits the release of catecholamine and blocks the sympathetic nervous system. Consequently, bradycardia may occur via the cardiovagal nervous system, but this adverse effect is easily managed in most cases by injection of drugs such as ephedrine or atropine.

We here present the case of a female patient with left anterior fascicular block who received spinal anesthesia for surgery, with the addition of intravenous dexmedetomidine, and experienced cardiac asystole after abrupt development of severe bradycardia.

## 2 Case presentation

**Figure 1:**
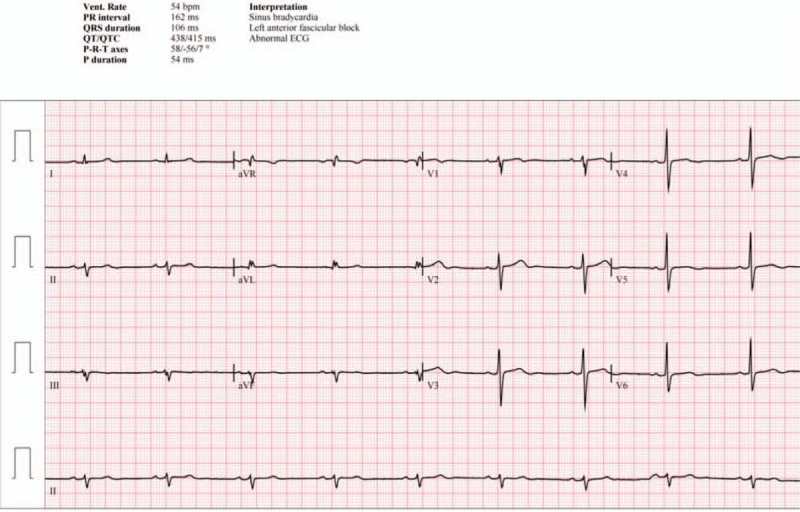
Preoperative electrocardiograph showing sinus bradycardia and left anterior fascicular block.

A 57-year-old female patient who had no underlying disease was admitted to our hospital for arthroscopic meniscus resection for a right meniscus tear. Preoperative electrocardiogram (ECG) showed a sinus bradycardia of 54 beats/min and left anterior fascicular block (Fig. 1). Cardiologists in our hospital assessed the risk of surgery and concluded that the patient was at low risk. The other preoperative findings were normal.

**Figure 2:**
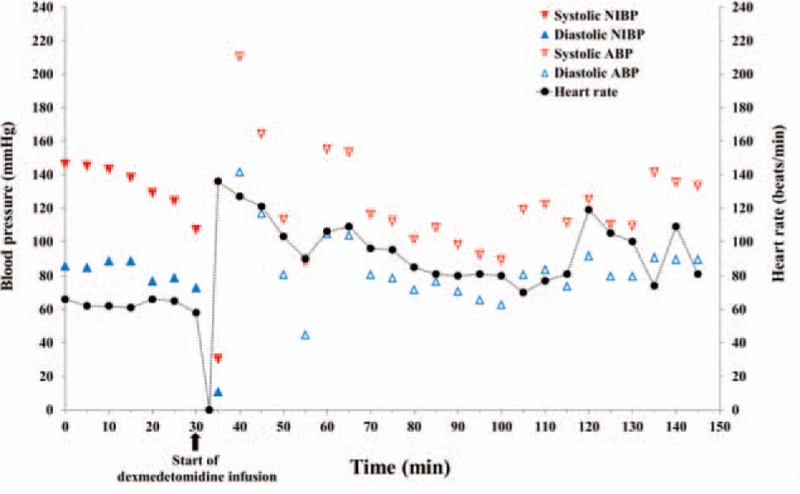
Flow chart of intraoperative vital signs during and after cardiopulmonary resuscitation. The black arrow indicates the time of initiation of dexmedetomidine infusion ABP = arterial blood pressure, NIBP = noninvasive blood pressure.

On the day of surgery, in the operating room, her measured vital signs were normal, with blood pressure of 146/86 mm Hg, heart rate of 66 beats/min, and SpO_2_ of 97%. In the right lateral decubitus position, the skin over the lumbar spine was sterilized with potadine, and 1% lidocaine 3 mL was injected at the level of L3–4, for subcutaneous anesthesia. After dural puncture using a 23-gauge Quincke needle via a median approach, the flow of clear cerebrospinal fluid was confirmed. After 11 mg of 0.5% heavy bupivacaine was injected through the spinal needle, she felt a sense of warmth in her lower extremities; her position was then changed to a supine position. A pinprick test was performed and the upper level of sensory loss was confirmed at T6. Dexmedetomidine infusion was planned at a loading dose of 1.0 mcg/kg/min over 10 minutes, followed by 0.7 mcg/kg/minutes intravenously, as a sedative. Thus, 20 minutes after heavy bupivacaine injection, injection of the loading dose of dexmedetomidine was commenced at a rate of 1 mcg kg^−1^ min^−1^ to sedate the patient. Two minutes after dexmedetomidine injection, the patient's heart rate had decreased to 31 beats/min and progressed to asystole within 10 seconds. Dexmedetomidine administration was stopped immediately. Mask ventilation and chest compressions were performed, along with intravenous injection of atropine (0.5 mg) and epinephrine (1 mg). Shortly after initiation of cardiopulmonary resuscitation (CPR), the cardiac rhythm returned to sinus rhythm and the femoral pulse became palpable. At that time, her heart rate was 130 beats/min (Fig. 2).

**Figure 3:**
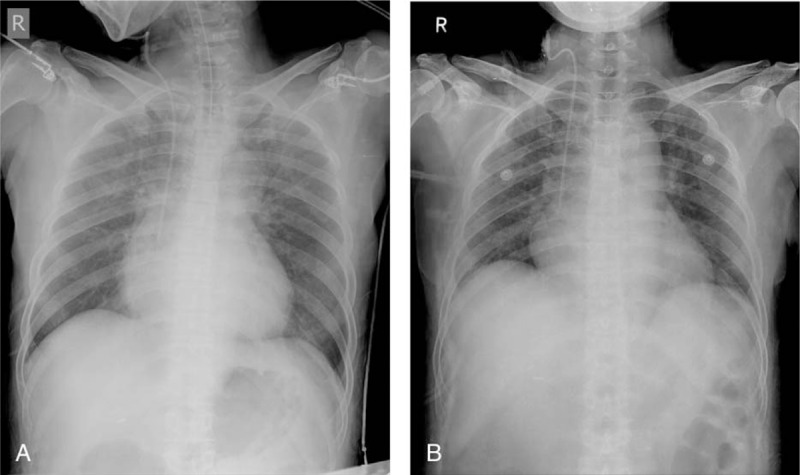
Postoperative pulmonary edema (A) was improved by the 4th postoperative day (B).

After radial artery catheterization, surgery was successfully performed under general anesthesia. Mild pulmonary edema was observed on the postoperative chest X-ray, but the patient recovered spontaneous respiration and left the operating room without mask ventilation. Four days later, pulmonary edema was improved (Fig. 3). During hospitalization, further evaluation of her cardiovascular function showed T-wave abnormalities on ECG and local wall motion abnormalities on echocardiography. No stenosis of the coronary arteries was seen in coronary angiography. Thus, these changes were considered to be typical of stress-induced cardiomyopathy, which often occurs after CPR. At 4 days after discharge, during which she experienced no abnormalities, the patient complained of intermittent dizziness. A 24-hour Holter ECG was then performed, but her average heart rate was 63 beats/min, with less than 3 premature ventricular contractions and premature atrial contractions. When she visited the cardiology outpatient clinic at 1 month and at 3 months after surgery, her symptoms had improved and she had no complaints.

## 3 Discussion

Dexmedetomidine is a selective α-2 agonist that is 8 times more potent than clonidine. It has the pharmacologic characteristics of a sedative and analgesic, decreasing the activity of the sympathetic nervous system, which helps to reduce the dose requirement of major anesthetics during surgery.^[2]^ Patients can be sedated without severe respiratory suppression and are easily awoken by stimulus. Therefore, the use of dexmedetomidine can be helpful for patients who are in the intensive care unit and on a ventilator.

Left bundle branch block (LBBB) is a cardiac conduction abnormality noted on ECG. In LBBB, activation of the left ventricle is delayed, such that the left ventricle contracts later than the right ventricle. Individuals with LBBB may still be quite active, presenting with no more than an abnormality on their ECG. However, when bundle branch blocks are complex and diffusely affect the bundle systems, or are associated with additional and significant ventricular muscle damage, they may be a sign of serious underlying heart disease. In more severe cases, a pacemaker may be required to restore an optimal electrical supply to the heart muscle. In some patients with LBBB, having a markedly prolonged duration of QRS complex (usually >150 milliseconds), a biventricular pacemaker that allows for better synchrony of heart contraction, may help to protect against systolic heart failure.^[3]^

Dexmedetomidine also has predictable cardiovascular inhibitory effects and it has been reported that up to 42% of patients experience adverse effects, such as hypotension and bradycardia.^[4]^ In healthy young subjects, the plasma concentration of norepinephrine decreases dose dependently and the release of norepinephrine reduces up to 92% after using dexmedetomidine.^[5]^ Therefore, administration of dexmedetomidine is not recommended for patients who are on medication for decreased cardiac function or left ventricular dysfunction. Hammer et al^[6]^ has observed ECG changes in children to whom dexmedetomidine was administered and found that it prolongs the sinus node recovery phase. This has adverse effects on both the sinoatrial (SA) and atrioventricular (AV) node. In previously published case reports about cardiac arrest occurring while using dexmedetomidine, the patients were mostly over 50 years old, and had preexisting cardiovascular disorders. Among those cases, Nagasaka et al^[7]^ reported that 1 patient with asymptomatic first-degree AV block experienced complete AV block during low anterior resection and eventually suffered cardiac arrest after dexmedetomidine administration.

In our case, the patient's preoperative ECG showed left anterior fascicular block and sinus bradycardia. This is usually regarded as a low risk for surgery when the patient has normal ventricular function and is asymptomatic. However, because dexmedetomidine can decrease the release of catecholamine in the body, the risk of developing severe bradycardia after using dexmedetomidine can be higher in patients with left anterior fascicular block and sinus bradycardia, even when they are asymptomatic, than in those who have normal sinus conduction. Moreover, in the presence of cardiac conduction disorders, such as left anterior fascicular block, SA and AV node dysfunction may decrease the electrical activity of the ventricles to such an extent that transient asystole can occur.

Spinal anesthesia blocks the sympathetic nervous system and induces vasodilatation of the anesthetized lower extremity. Hence, it reduces the venous return to the heart, which can lead to adverse effects on the cardiovascular system. With total spinal anesthesia, respiratory function as well as cardiac function is suppressed. However, reviews of cases of patients who underwent cardiac arrest during spinal anesthesia and epidural anesthesia revealed that the upper level of sensory loss ranged from T5 to T10, and that there was no correlation between the use of fluid replacement and cardiac arrest. Furthermore, the onset of cardiac arrest varied widely, from 5 to 180 minutes, after injection of the local anesthetic.^[8]^ In our case, the upper level of the loss of sensation after spinal anesthesia was at T6. Prophylactic fluid replacement was performed before complete spinal block and the level of anesthesia was fixed before dexmedetomidine was administered. Thus, sufficient time was given for body fluid compensation, and there was no evidence that the probability of cardiac arrest had increased due to spinal anesthesia alone. In patients with a basal heart rate of less than 60 beats/min, the possibility of bradycardia during spinal anesthesia was 5 times higher than in normal patients, and bradycardia was 3 times more frequent in patients of American Society of Anesthesiologists’ physical status 1, with hyperactive vagus nervous function.^[9]^ The present case could therefore be considered as having a greater risk of severe bradycardia during spinal anesthesia, as she had sinus bradycardia preoperatively. However, she showed no signs of worsening bradycardia after anesthesia until the range of the nerve block had somewhat stabilized. Therefore, the likelihood that cardiac arrest occurred only due to spinal anesthesia, irrespective of dexmedetomidine administration, was considered low.^[10]^

Previously published case reports of cardiac arrest associated with dexmedetomidine have involved administration of dexmedetomidine in addition to intravenous anesthetics or inhalation anesthetics during general anesthesia. This case emphasized that asystole could occur with continuous infusion of low-dose dexmedetomidine even after spinal anesthesia, in a patient with left anterior fascicular block and sinus bradycardia, likely due to the sympathetic inhibitory effect of dexmedetomidine. In patients with sinus bradycardia and anterior fascicular block, administration of dexmedetomidine requires close monitoring for a hyperactive parasympathetic nervous system response, such as in individuals receiving spinal anesthesia.
